# Cryoprobe‐Assisted Bronchoscopy: Noninvasive Route to Definitive Diagnosis of Pulmonary Lymphomatoid Granulomatosis

**DOI:** 10.1155/crpu/6543133

**Published:** 2026-04-07

**Authors:** Youjin Kim, Thomas C. Landry, Nicholas Wysham

**Affiliations:** ^1^ Department of Medicine, Legacy Salmon Creek Medical Center, Vancouver, Washington, USA; ^2^ Department of Pulmonology, The Vancouver Clinic, Vancouver, Washington, USA

**Keywords:** bronchoscopy, case report, cryoprobe biopsy, Epstein–Barr virus, lung nodules, pulmonary lymphomatoid granulomatosis

## Abstract

Pulmonary lymphomatoid granulomatosis (PLG) is a rare Epstein–Barr virus (EBV)–driven B‐cell lymphoproliferative disorder characterized by angiocentric and angiodestructive infiltrates. The disease predominantly affects middle‐aged immunocompromised males, with a median survival of 14 months and mortality rates of 63%–90% at 5 years. Histopathological evaluation remains the cornerstone of diagnosis, historically requiring surgical lung biopsy for adequate tissue acquisition. We present a novel case of an 82‐year‐old male with a history of COPD and colon cancer who developed rapidly progressive, predominantly right upper lobe pulmonary nodules. The patient′s initial presentation with fever and hypotension led to an inpatient workup for sepsis, where his lung nodules were incidentally noted to have grown. Following referral to pulmonology, a robotic navigation bronchoscopy (ion) with a cryoprobe (Erbe) was performed for tissue diagnosis, initially considering metastatic disease. Pathology from the cryoprobe biopsy yielded a consensus diagnosis of Grade 3 PLG after expert review. This case raises awareness of a rare diagnosis in the differential of progressive pulmonary nodules. It also demonstrates an atypical presentation with the patient′s advanced age, the aggressive and unusual distribution of the nodules, and the absence of classic extrapulmonary manifestations. Most importantly, it demonstrates that a novel, less invasive, cryoprobe‐assisted bronchoscopic biopsy can be sufficient for a definitive diagnosis of this rare disease, a modality not previously reported for PLG diagnosis, potentially precluding the need for more invasive surgical procedures.

## 1. Introduction

Epstein–Barr virus is a ubiquitous human herpes virus known to be associated with a wide spectrum of lymphoproliferative disorders and various malignancies. These EBV‐associated lymphoproliferative disorders are a heterogeneous group characterized by harboring latent EBV within tumor cells, and immunodeficiency states can increase the risk of their development [1, 2]. Pulmonary lymphomatoid granulomatosis (PLG) is recognized as a rare, angiocentric and angiodestructive EBV‐driven B‐cell lymphoproliferative disorder, characterized by a polymorphous infiltrate containing atypical B cells in a T‐cell rich background [1, 3]. It is also specifically categorized as one of the rare subtypes of EBV‐associated B‐cell lymphoproliferative disorders. It is hypothesized that patients with PLG have dysregulated immune surveillance of EBV, leading to the proliferation of EBV‐infected B cells [1, 4]. The World Health Organization classifies PLG under “mature B‐cell neoplasms,” and it is hypothesized that patients with PLG have dysregulated immune surveillance of EBV, leading to the proliferation of EBV‐infected B cells [1, 4]. Although primarily affecting the lungs, it exhibits the potential for extrapulmonary involvement.

PLG is a rare condition predominantly affecting middle‐aged (40–70 years), immunocompromised males (male − to − female ratio < 2 : 1) experiencing nonspecific pulmonary symptoms. Known risk factors include history of EBV infection, underlying immunodeficiency (HIV and Wiskott–Aldrich syndrome) states, use of immunosuppressive medications (notably methotrexate and azathioprine), and organ transplantation [1, 5, 6]. This disease primarily affects the lungs in over 90% of patients but exhibits potential for extrapulmonary involvement of the skin, central and peripheral nervous system, kidneys, and liver [3, 5]. Characteristic presentations include nonspecific pulmonary symptoms such as cough, dyspnea, and hemoptysis, along with constitutional symptoms including fever, weight loss, and night sweats. Extrapulmonary manifestations may include skin rashes and neurological deficits such as ataxia and cranial nerve disorders [2, 4, 5]. Imaging studies typically reveal multiple pulmonary nodules, frequently situated in the mid to lower lobes, which can display a waxing and waning progression. Overall prognosis remains poor, with median survival of approximately 14–24 months from diagnosis and mortality rates of 60%–90% at 5 years, though the clinical course is variable with reports of prolonged courses and rare spontaneous remission [3, 6, 7]. Poor prognostic indicators include central nervous system involvement, Grade 3 histology, young age at diagnosis (< 25 years), leukocytosis, and hepatosplenomegaly [6, 7].

Historically, definitive histological diagnosis, reliant on assessing angioinvasion and grading, has traditionally mandated surgical lung biopsy for adequate tissue acquisition [3]. Traditionally, this has mandated surgical lung biopsy either video‐assisted thoracoscopic surgery (VATS) or open thoracotomy to obtain adequate tissue for histopathological evaluation [3, 8]. However, these surgical approaches carry significant morbidity, require general anesthesia with single‐lung ventilation, and are contraindicated in patients with severe respiratory impairment. CT‐guided percutaneous transthoracic needle biopsy offers an alternative with high diagnostic yield (80%–90%), but is associated with substantial complication rates, including pneumothorax in 15% of cases and chest tube placement up to 6.6% [9]. Robotic‐assisted bronchoscopy (RAB) with transbronchial cryobiopsy has emerged as a promising minimally invasive alternative, offering diagnostic yields comparable with CT‐guided biopsy (82%–88%) with significantly reduced complication rates (3%–4%) [10, 11].

This paper describes the case of an 82‐year‐old male who presented with an atypical, rapidly progressive constellation of predominantly right upper lobe pulmonary nodules. A diagnosis of Grade 3 PLG was conclusively achieved through a novel cryoprobe‐assisted bronchoscopic biopsy. This presentation emphasizes the critical importance of considering PLG within a broad differential diagnosis and demonstrates the substantial diagnostic utility of advanced bronchoscopic techniques in replacing more invasive surgical procedures.

## 2. Case Report

An 82‐year‐old male with a history of COPD, Stage 2 colon cancer status postresection (3/2017), and a 16 pack‐year smoking history presented to the emergency department on March 27, 2023, with a several‐month history of chronic cough, new‐onset fever, and hypotension. On admission, the patient was found to be in atrial fibrillation with rapid ventricular response. He had a hemoglobin of 10.3 g/dL and leukocytosis of 11.9 × 10^9^/L. A chest x‐ray revealed nodular opacities in the right upper lobe. A noncontrast chest CT confirmed predominantly right‐sided pulmonary nodules, with the largest in the right lower lobe measuring 15 mm (increased from 12 mm 3 months prior). New nodules were also noted in the right upper lobe (10.3 mm, 9.2 mm, 5 × 5 mm) compared with a 2017 exam. A CT abdomen and pelvis with contrast was benign with no lymphadenopathy. A lung ventilation–perfusion scan was negative for pulmonary embolism despite a significantly elevated D‐dimer of 8.11. The patient was treated for sepsis of unknown source and discharged with a referral to pulmonology for evaluation of the nodules.

A retrospective review of the patient′s medical records revealed that incidental pulmonary nodules were first noted in February 2022 on a CT performed for dyspnea, measuring up to 6 mm. An outpatient follow‐up chest CT on March 10, 2023, prior to his admission, had already shown progression in the size and number of these nodules.

**Figure 1:**
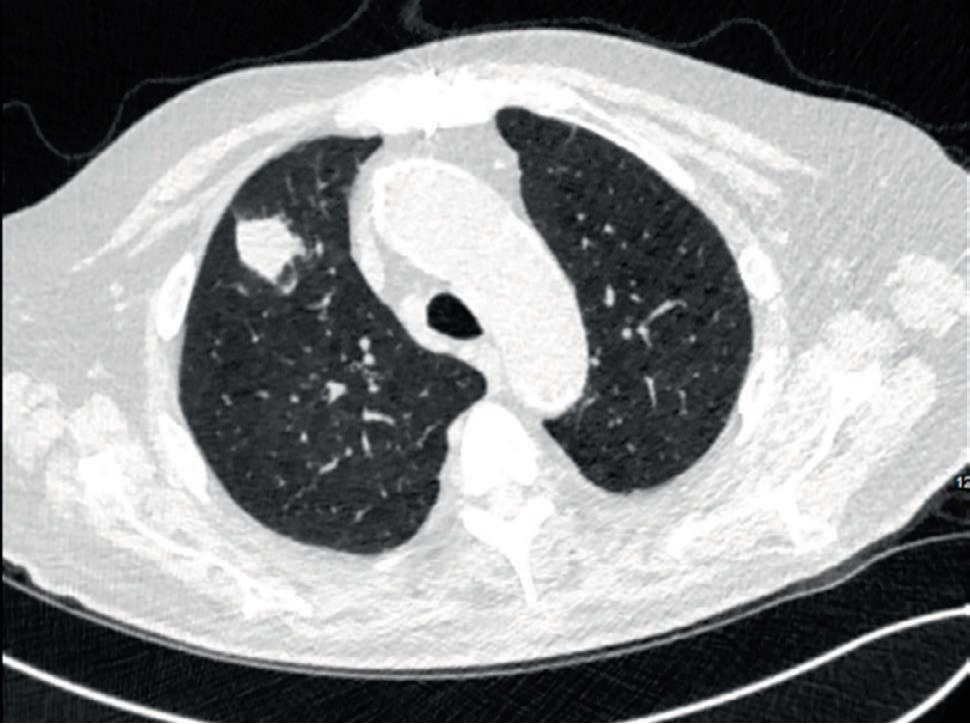
Noncontrast chest CT on April 11, 2023 showing right upper lobe nodules measuring 2.7 × 2.1 cm.

**Figure 2:**
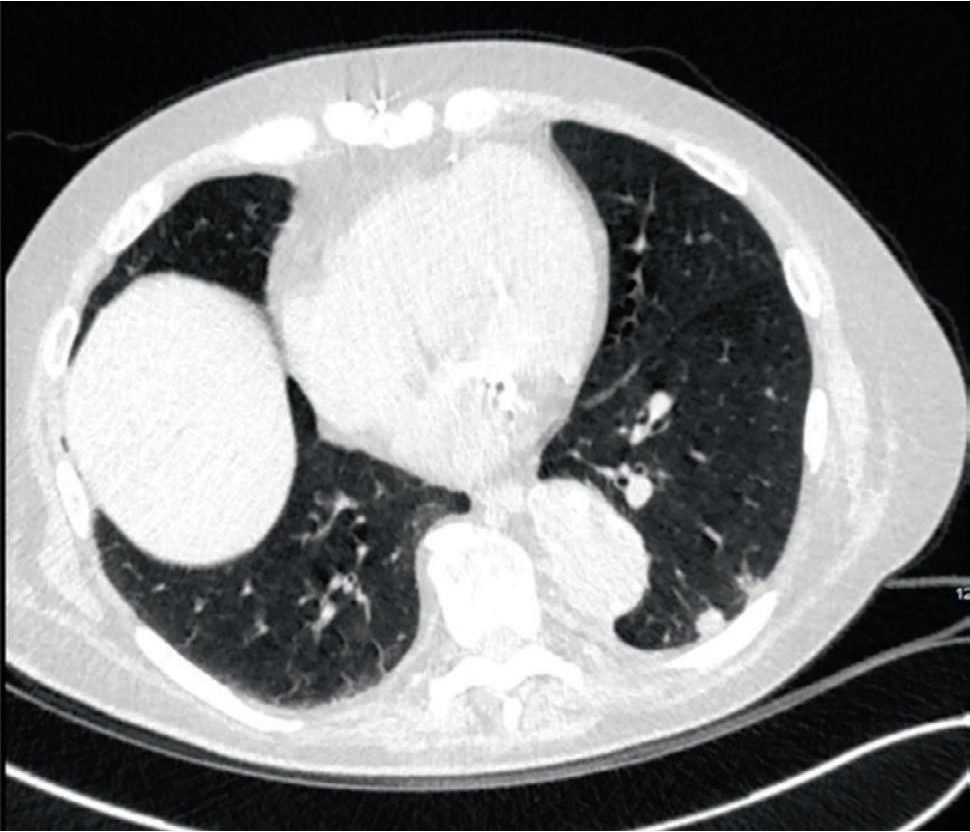
Noncontrast chest CT on April 11, 2023 showing an irregular nodule in the right lung apex measured 13 × 10 mm.

Upon outpatient follow‐up, a repeat noncontrast chest CT on April 11, 2023, showed continued rapid enlargement of the multiple pulmonary nodules over the course of 14 months. The largest anterior right upper lobe nodule measured 2.7 × 2.1 cm, and an irregular nodule in the right lung apex measured 13 × 10 mm (Figures 1 and 2). Given the rapid growth and the patient′s history, the working differential was metastatic colon cancer or primary lung cancer.

On April 12, 2023, the patient underwent robotic navigation bronchoscopy (ion) for a tissue diagnosis. Biopsies were guided with fluoroscopy and radial endobronchial ultrasound (rEBUS) and obtained from the right upper lobe lesion using fine needle aspiration, forceps, and a 1.1‐mm cryoprobe (Erbe Elektromedizin GmbH). The cryoprobe technique employed freeze times of 3–5 s using a “cloud technique” approach with sampling from five angles to maximize diagnostic yield and minimize the risk of missing the most diagnostic area of the lesion.

**Figure 4:**
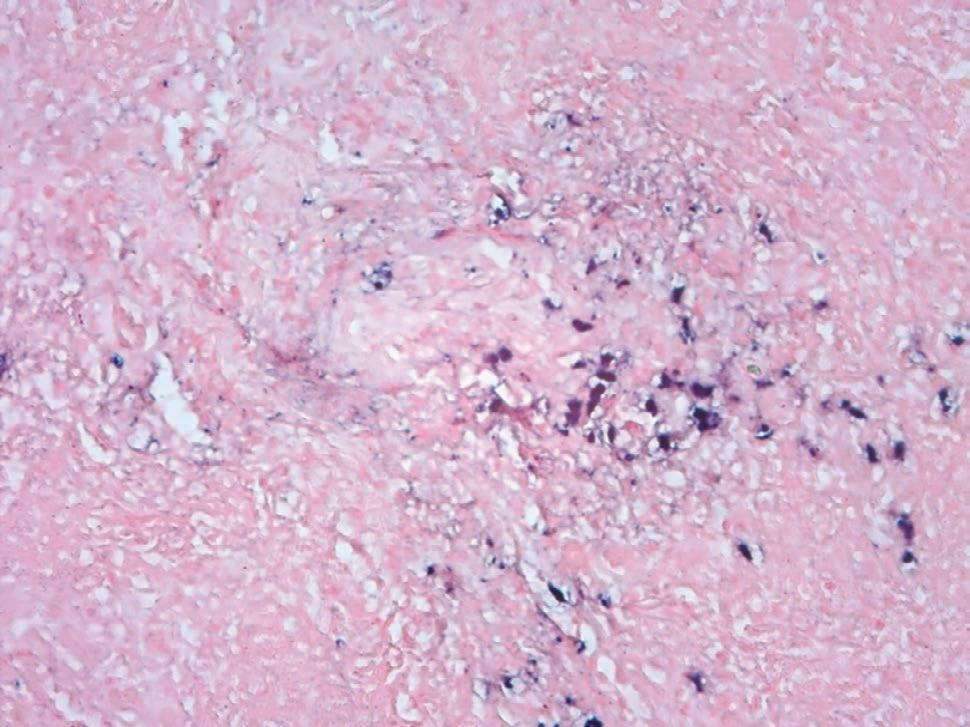
Histology slide demonstrating EBV‐encoded RNA (EBER) in situ hybridization in ×200 power.

Histopathological examination on April 19, 2023, confirmed a diagnosis of PLG, Grade 3. Notably, the cryobiopsy specimens demonstrated excellent tissue preservation without significant crush artifact, enabling clearing visualization of the architectural features essential for diagnosis (Figure 3a). The biopsies revealed a necrotic lymphoid lesion with atypical lymphoid cells and focal invasion of vessel walls (Figure 3b). Immunohistochemistry showed positive staining for CD20, PAX5 (demonstrating predominant atypical B‐cell infiltrate, Figure 4) with reactive T cells highlighted by CD3 (Figure 5a). EBV in situ hybridization (EBER) was positive (Figure 5b), and Ki‐67 expression of 40%–50%, consistent with a high‐grade lymphoproliferative disorder. No infectious etiology was identified.

Figure 3(a) Lowest power (×25) of the specimen demonstrating well‐preserved tissue architecture without crush artifact on the left. (b) Higher power (×200) image on the right highlighting the atypical lymphoid cells and the characteristic angiodestructive growth patterns.

**Figure:**
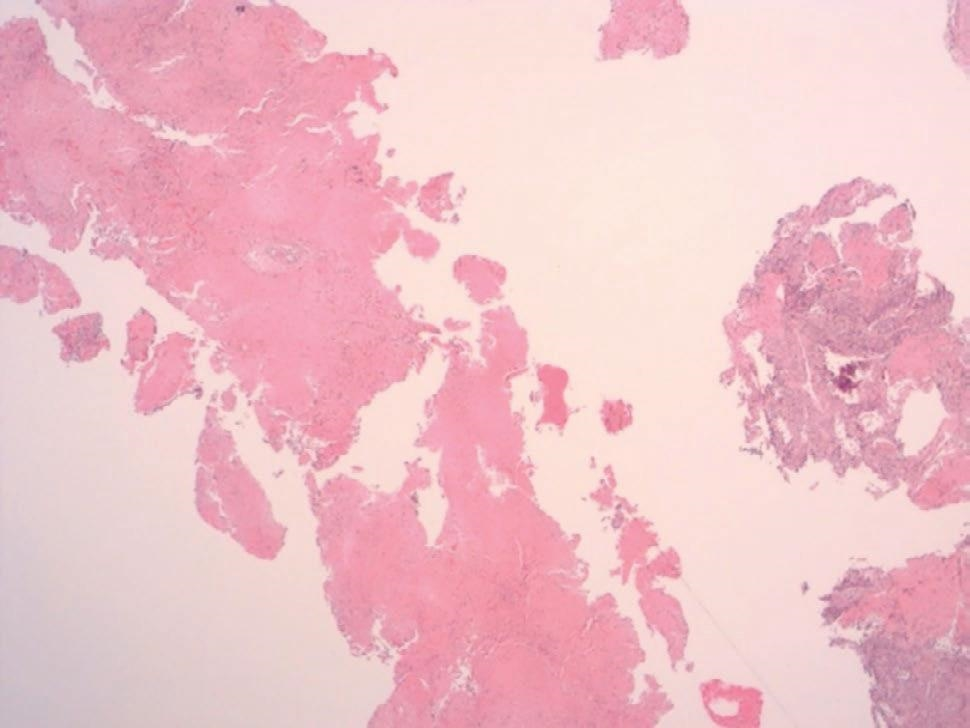
(a)

**Figure:**
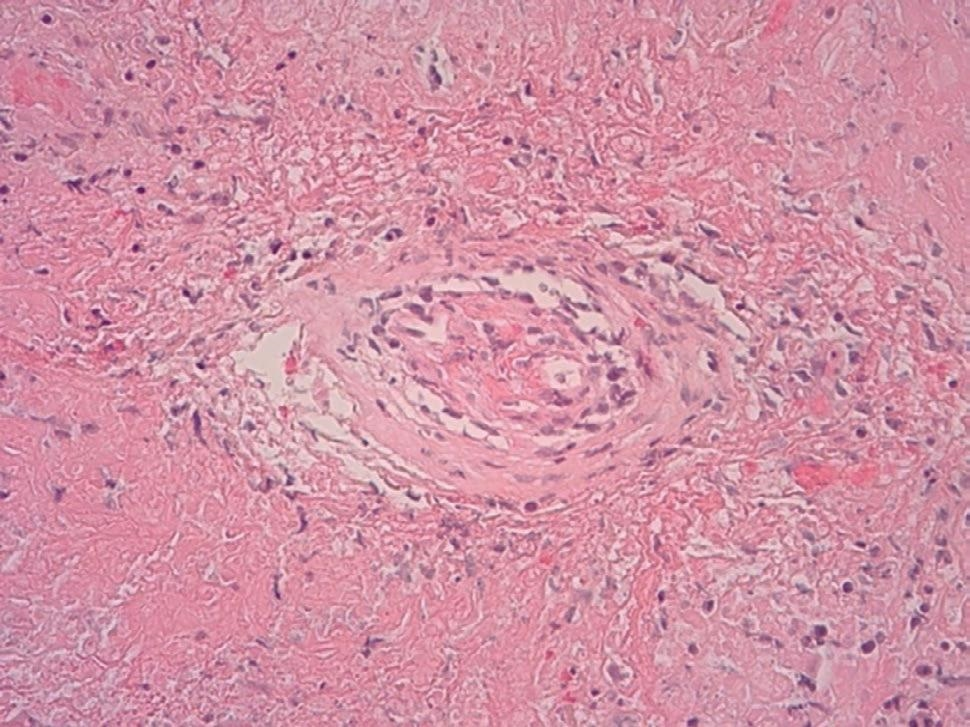
(b)

Figure 5(a) Histology slide with CD 20 immunostain on the left demonstrating predominant, atypical B‐cell infiltrate in ×100 power. (b) Histology slide with CD3 immunostain on the right highlighting reactive T cells in ×100 power.

**Figure:**
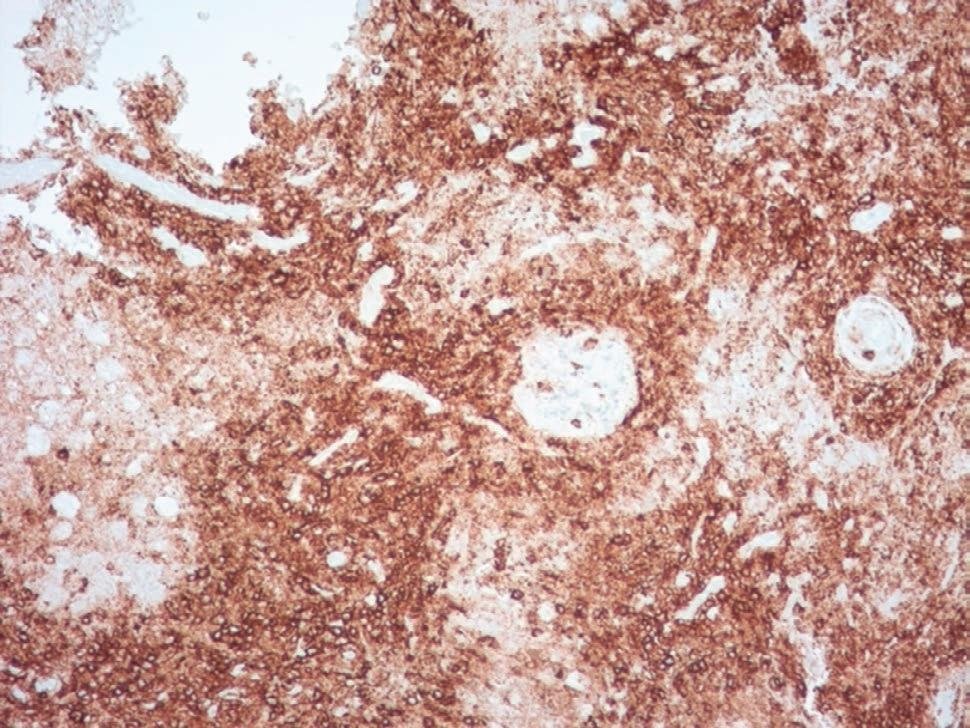
(a)

**Figure:**
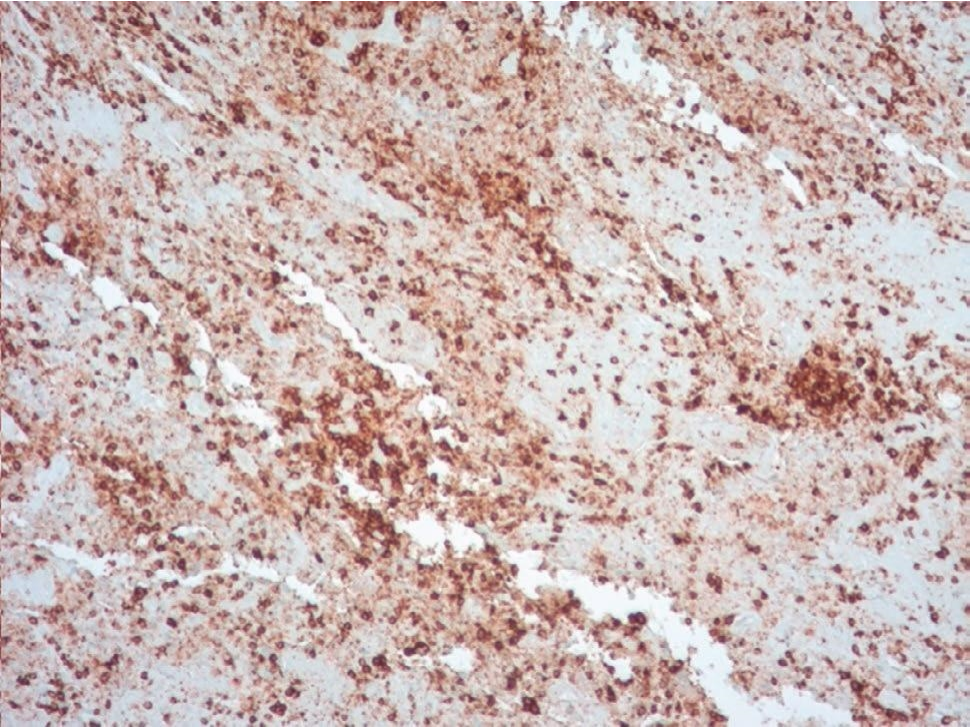
(b)

Throughout his clinical course, the patient did not exhibit classic extrapulmonary manifestations of PLG, such as skin rashes or new‐onset focal neurological deficits. Unfortunately, before specific therapy could be initiated, the patient′s condition rapidly declined, and he passed away at home from uncertain causes, not thought to be related to his PLG.

## 3. Discussion

This case highlights several important atypical features of PLG that expand our understanding of this rare disease′s clinical spectrum. First, although PLG typically affects middle‐aged men, this patient′s advanced age of 82 years underscores that age alone should not preclude its consideration, especially given that clinicians might prematurely dismiss it in an older demographic [3]. This is clinically significant because clinicians might prematurely dismiss PLG in an older demographic, particularly when more common malignancies such as metastatic disease or primary lung cancer are high on the differential. Second, the aggressive, rapid progression of the nodules over 14 months with unusual predominance in the right upper lobe created a significant diagnostic challenge by mimicking more common malignancies such as metastatic colon cancer or primary lung cancer. This distribution is atypical, as PLG classically presents with bilateral lower lobes predominant nodules that demonstrate a waxing and waning pattern [6]. Furthermore, the absence of the characteristic waxing and waning pattern, combined with the lack of classic extrapulmonary symptoms (skin rashes and neurological deficits) despite a high‐grade pathology, further complicated the diagnostic process, diverting attention from PLG towards more typical presentations of neoplastic or infectious diseases [12].

Given the diagnostic challenges associated with PLG, clinicians should maintain a high index of suspicion for this rare disease in patients presenting with progressive pulmonary nodules or masses, particularly those with known immunodeficiency, prior EBV infection, or use of immunosuppressive medications. Clinical features that should raise suspicion for PLG include multiple bilateral pulmonary nodules (especially with cavitation), a waxing and waning clinical course, concurrent skin lesions or neurological symptoms, and failure to respond to conventional antimicrobial or anti‐inflammatory therapies [7, 13]. Radiologically, PLG may present with ground‐glass opacities, consolidations, or nodules that may demonstrate cavitation, and PET‐CT typically shows FDG‐avid lesions [14].

The most critical learning point from this case is the successful use of a less invasive diagnostic modality for a disease traditionally thought to require surgical lung biopsy. The choice of biopsy modality for pulmonary nodules depends on lesion characteristics, patient factors, institutional expertise, and available technology. VATS and open thoracotomy are the usual biopsy modality which are invasive surgical procedures requiring general anesthesia, single‐lung ventilation, and hospitalization with associated morbidity that may be prohibited in elderly or comorbid patient although they can provide large tissue samples [8]. CT‐guided percutaneous transthoracic needle biopsy offers high diagnostic yield but carries significant complication risks, with pneumothorax and chest tube requirements [9, 10]. Recent advances in RAB have demonstrated comparable diagnostic yield (82%–88%) with significantly reduced complication rates (3%–4%), making it an attractive alternative for peripheral pulmonary lesions [10, 11, 15].

Transbronchial lung cryobiopsy (TBCB) is an emerging technique proposed as an alternative to surgical lung biopsy due to its high diagnostic yield and good safety profile [16]. Studies have shown TBCB can achieve diagnostic accuracy rates of up to 95% for endobronchial tumors and approximately 90% for peripheral pulmonary nodules [17–19]. Compared with traditional transbronchial forceps biopsy, cryobiopsy obtains larger tissue samples with superior preservation of histological architecture and minimal crush artifact, facilitating accurate pathological assessment [8, 16, 19]. Studies have demonstrated that a 1.1‐mm cryoprobe biopsy had a better diagnostic yield of 84.6% compared with standard which was 69.2% with minimal complications [20]. The larger, better‐preserved specimens obtained through cryobiopsy are particularly valuable for diagnosing complex lymphoproliferative disorders like PLG. In our case, the cryobiopsy specimens clearly demonstrated the angiodestructive features, atypical lymphoid cells, and EBC positivity essential for the diagnosis of Grade 3 PLG which may have been compromised or uninterpretable with smaller, crushed‐affected forceps biopsy specimens.

The combination of robotic bronchoscopy with cryobiopsy leverages the advantages of both technologies. Robotic platforms such as ion endoluminal system provide precise navigation to peripheral lesions that may be inaccessible with conventional bronchoscopy, whereas a 1.1‐mm cryoprobe with an oversheath enhances safety by allowing the procedure to be performed without removing the bronchoscope from the airway [20, 21]. The cloud technique minimizes the risk of sampling error and maximizes diagnostic yield [17, 21]. Additionally, robotic bronchoscopy allows for simultaneous mediastinal staging via endobronchial ultrasound–transbronchial needle aspiration (EBUS‐TBNA) in a single procedure, which may be valuable in cases where lymphoma is suspected [22].

This case demonstrates the expanding role of cryoprobe bronchoscopy to replace more invasive surgical procedures for the diagnosis of complex and rare pulmonary diseases, reducing patient morbidity and the time to diagnosis. Given advancements in cancer therapies requiring maximum tissue volume for genotyping and phenotyping, TBCB is increasingly considered a first‐line tool for lung cancer diagnosis [23–25]. However, it is crucial to note that patient selection for cryosurgical procedures must be individualized, considering potential perioperative risks, and these complex procedures should be carried out in specialized centers by trained and experienced teams.

## 4. Conclusion

PLG should be included in the differential diagnosis for rapidly progressive, atypical pulmonary nodules in elderly patients, even without classic clinical or radiological features. This case highlights that cryoprobe‐assisted robotic bronchoscopy can achieve definitive diagnosis of this rare and aggressive disease, providing larger, artifact‐free tissue samples sufficient for accurate pathological grading, including critical features of angioinvasion and EBV positivity. Compared with surgical lung biopsy and CT‐guided percutaneous biopsy, robotic bronchoscopy with cryobiopsy offers a favorable safety profile with reduced morbidity, potentially obviating the need for more invasive procedures. This case powerfully demonstrates the expanding role of advanced bronchoscopic techniques in replacing more invasive surgical procedures for the diagnosis of complex and rare pulmonary diseases, reducing patient morbidity and accelerating time to diagnosis.

## Funding

No funding was received for this manuscript.

## Consent

Consent was obtained. Given that the patient has passed away, verbal consent was obtained from next of kin.

## Conflicts of Interest

The authors declare no conflicts of interest.

## Data Availability

Data sharing is not applicable to this article as no datasets were generated or analyzed during the current study.
